# Sustained multisite response to anifrolumab in refractory Chilblain lupus erythematosus

**DOI:** 10.1016/j.jdcr.2026.08.051

**Published:** 2026-09-06

**Authors:** Sarah John, Lisabeth V. Scalzi, Galen Foulke, Matthew Helm

**Affiliations:** a Penn State College of Medicine, Hershey, Pennsylvania; b Division of Pediatric Rheumatology, Department of Pediatrics, Golisano Children’s Hospital, Penn State Health, Hershey, Pennsylvania; c Department of Dermatology, Penn State Health, Hershey, Pennsylvania; d Department of Public Health Sciences, Penn State Health, Hershey, Pennsylvania

**Keywords:** anifrolumab, chilblain lupus erythematosus, cutaneous lupus erythematosus, refractory disease, type I interferon

## Introduction

Chilblain lupus erythematosus (CHLE) is a rare variant of chronic cutaneous lupus erythematosus, closely associated with systemic lupus erythematosus (SLE) and other lupus-spectrum connective tissue diseases. It presents with cold-induced, pruritic or painful violaceous lesions on acral sites that may ulcerate, fissure, become hyperkeratotic, or form atrophic plaques.^1^

Management of CHLE focuses on cold avoidance and pharmacologic therapy, including oral or topical corticosteroids and calcium channel blockers. Antimalarials, onabotulinum toxin A, oral and IV prostaglandins, and Janus kinase inhibitors are often used in refractory cases; however, efficacy is variable and adverse effects may limit use.^1^^,^^2^ Growing evidence implicates dysregulated type I interferon (IFN) signaling in CHLE pathogenesis and provides a potential avenue for targeted therapy.^2^^,^^3^

Here, we report a case of severe, refractory CHLE with a sustained multisite cutaneous response to anifrolumab.

## Case report

A 30-year-old woman with a longstanding history of SLE presented to dermatology in November 2024 for worsening CHLE, characterized by painful acral lesions that worsened with cold exposure. She fulfilled 2019 EULAR/ACR classification criteria for SLE, including biopsy-proven class V lupus nephritis, mucocutaneous involvement (malar and discoid rashes, oral ulcers), inflammatory arthritis, and SLE-specific autoantibodies (anti-double-stranded DNA and anti-Smith). Additional findings included anti-ribonucleoprotein and antiphospholipid antibodies, hypocomplementemia, and Raynaud phenomenon.^4^ She also had a family history notable for 2 siblings with SLE.

Despite ongoing therapy, her CHLE remained severe and refractory for over 5 years to multiple treatments, including systemic and topical corticosteroids, pentoxifylline, intravenous prostaglandins, vasodilators (nifedipine, sildenafil), mycophenolate mofetil, and intermittent botulinum toxin injections.

In late October 2024, she experienced a severe SLE flare complicated by worsening CHLE and a secondary infection with methicillin-resistant *Staphylococcus aureus* and *Escherichia coli* on the hands. Given the severity of her disease, she was admitted and treated with intravenous (IV) antibiotics, IV methylprednisolone, and two 1-gram rituximab infusions. Belimumab was discontinued at the time of rituximab infusion. While partial improvement was noted following hospitalization and rituximab administration, the patient continued to have persistent and severe CHLE. The patient's clinical course and treatment timeline are summarized in Fig 1.

**Fig 1:**
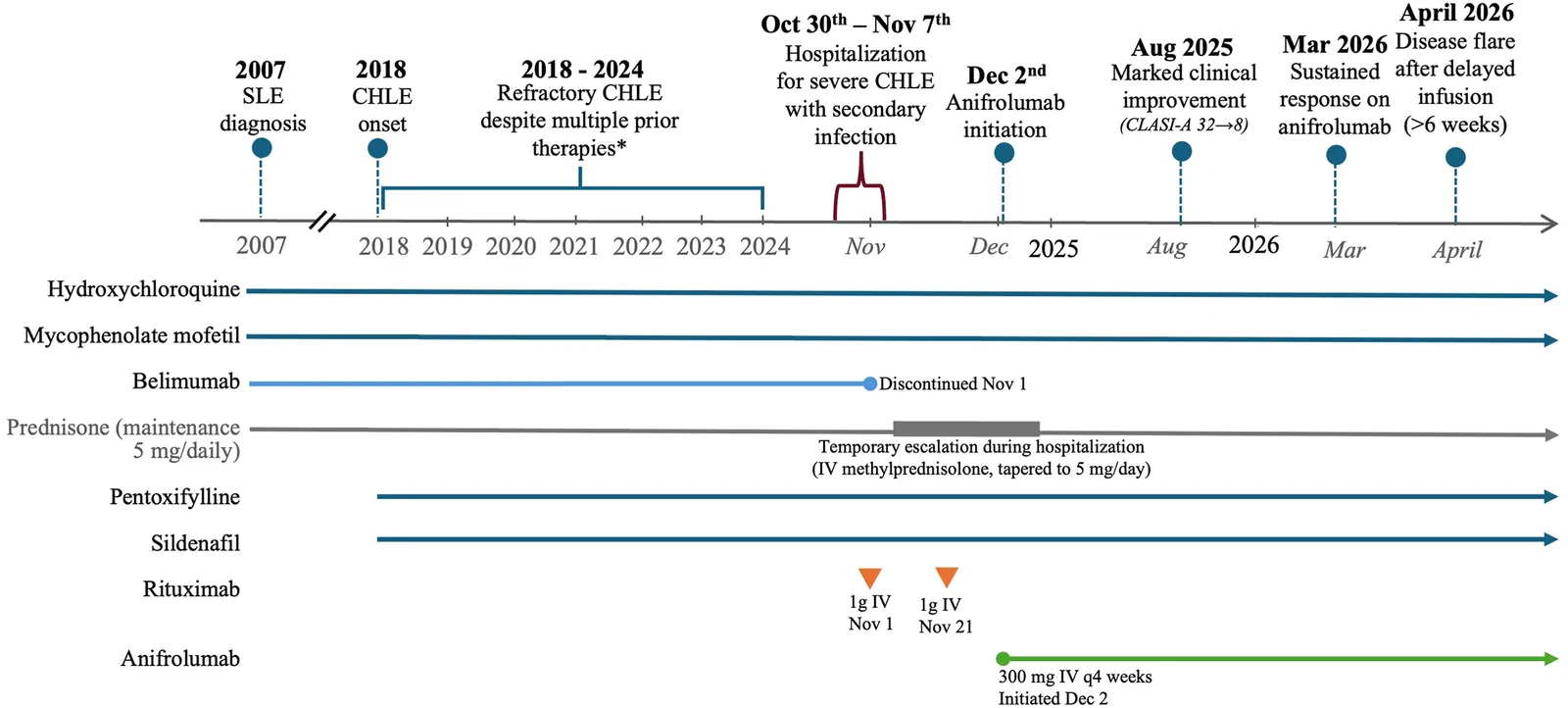
Timeline demonstrating the patient's clinical course of CHLE and medication exposure before and after initiation of anifrolumab. Hydroxychloroquine, mycophenolate mofetil, pentoxifylline, and sildenafil were continued throughout treatment. Belimumab was discontinued during hospitalization before anifrolumab initiation. During hospitalization, prednisone was escalated with intravenous methylprednisolone and tapered to 5 mg daily. Rituximab (1 g) was administered on November 1 and November 21, 2024, and anifrolumab was initiated on December 2, 2024. ∗Prior therapies included topical corticosteroids, nifedipine, intravenous prostaglandins, and botulinum toxin injections.

At her November 2024 follow up, physical examination revealed erythematous, desquamative patches with erosions, skin induration, and narrowing of the distal digits (Figs 2, *A* and 3, *A*), associated with severe pain (10/10) and impaired hand function. Progressive patchy scarring alopecia of the scalp was also noted, consistent with cutaneous lupus (Fig 4, *A*). Radiographs demonstrated fingertip soft tissue atrophy, and genetic testing for inherited type I interferonopathies, including *TREX1, SAMHD1, RNASEH2A/B/C,* and *STING1,* was negative (Supplementary Appendix, available via Mendeley at https://data.mendeley.com/datasets/78yp6vt6mh/1).

**Fig 2:**
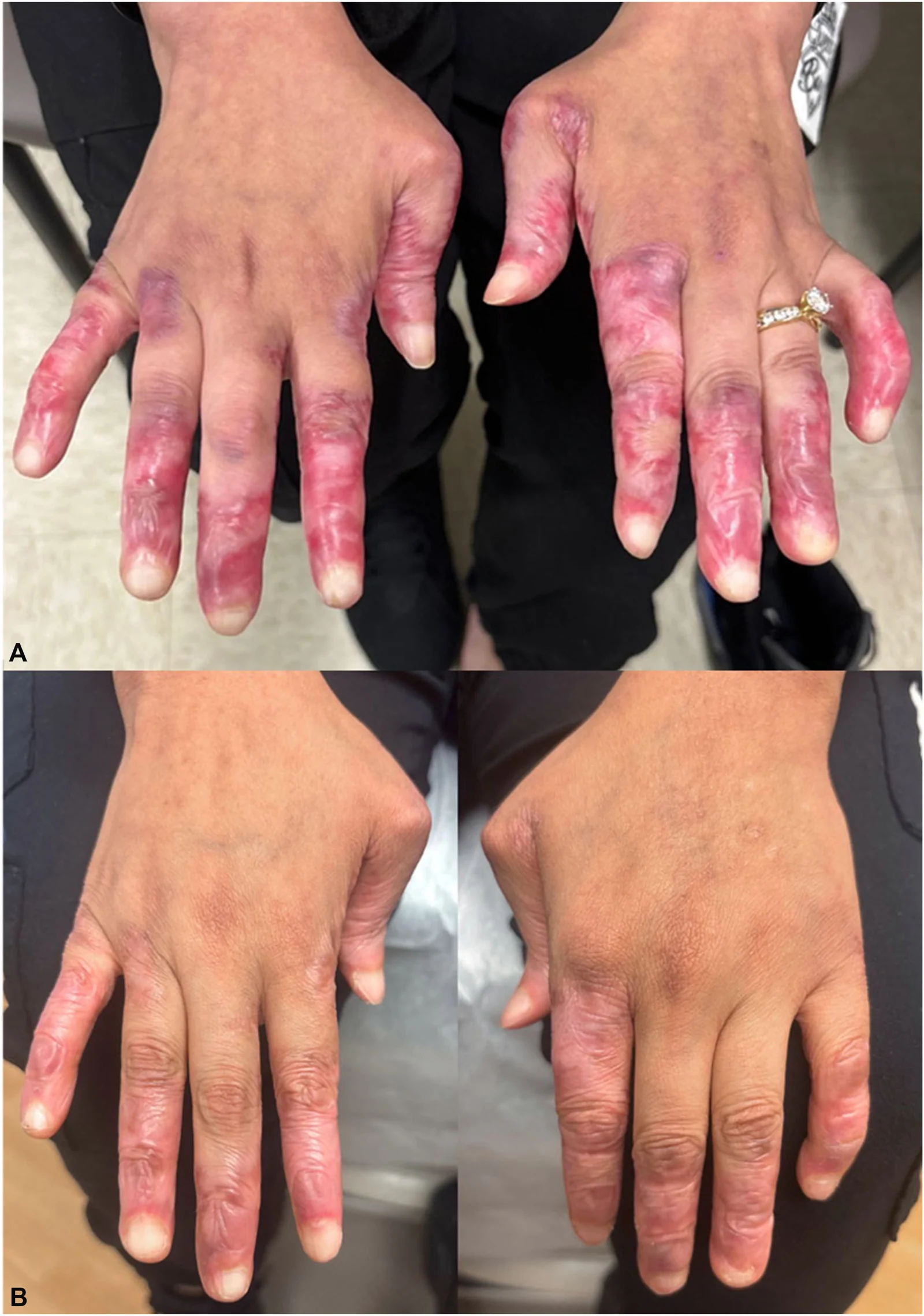
CHLE affected hands before and after anifrolumab. **A,** Erythematous, desquamative patches on the fingers with fingertip erosion. **B,** Visible improvement in digital lesions after 7 months of anifrolumab infusions.

**Fig 3:**
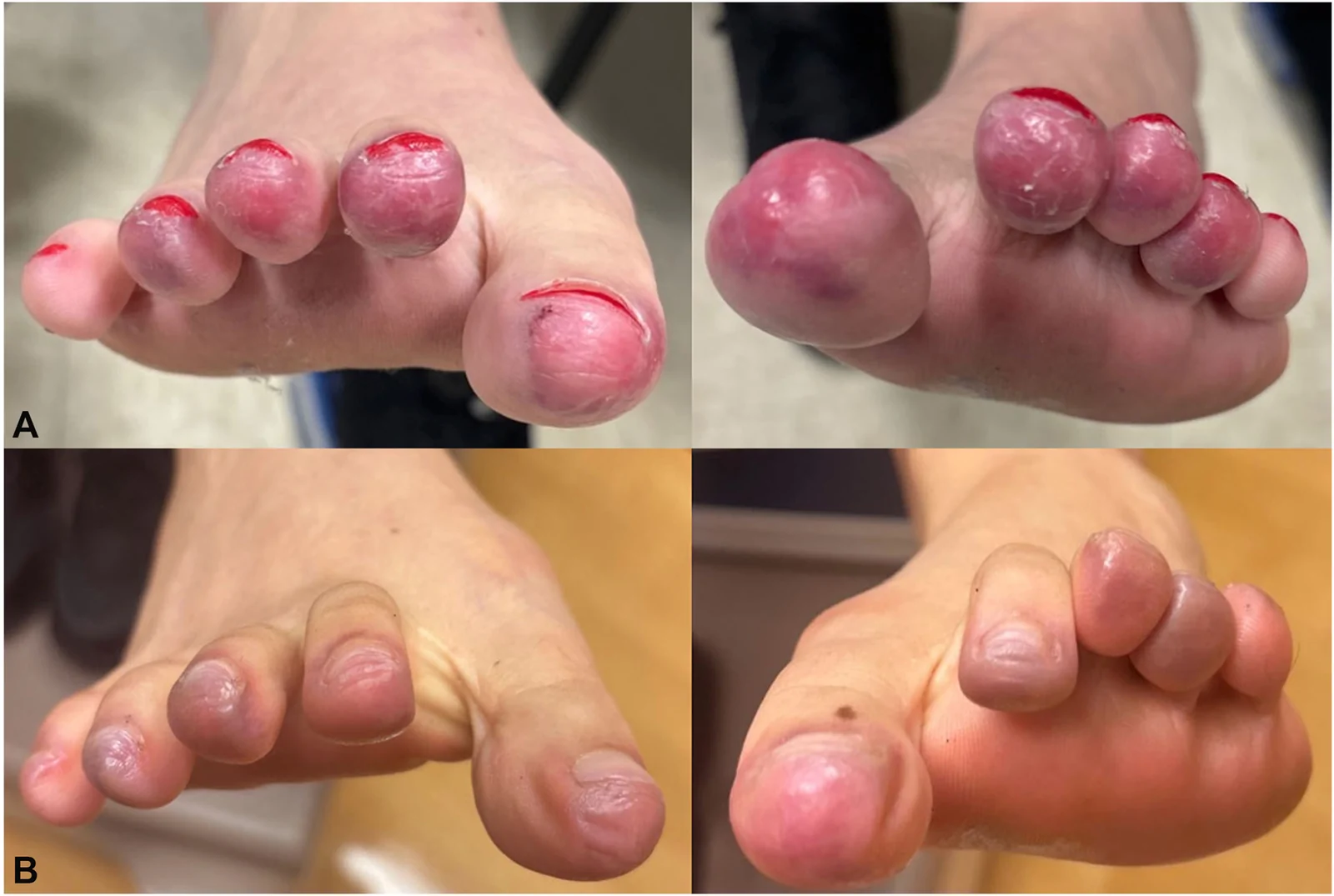
CHLE of the feet before and after anifrolumab therapy. **A,** Erythematous, desquamative patches involving the toes. **B,** Significant improvement of acral lesions after 7 months of anifrolumab infusions.

**Fig 4:**
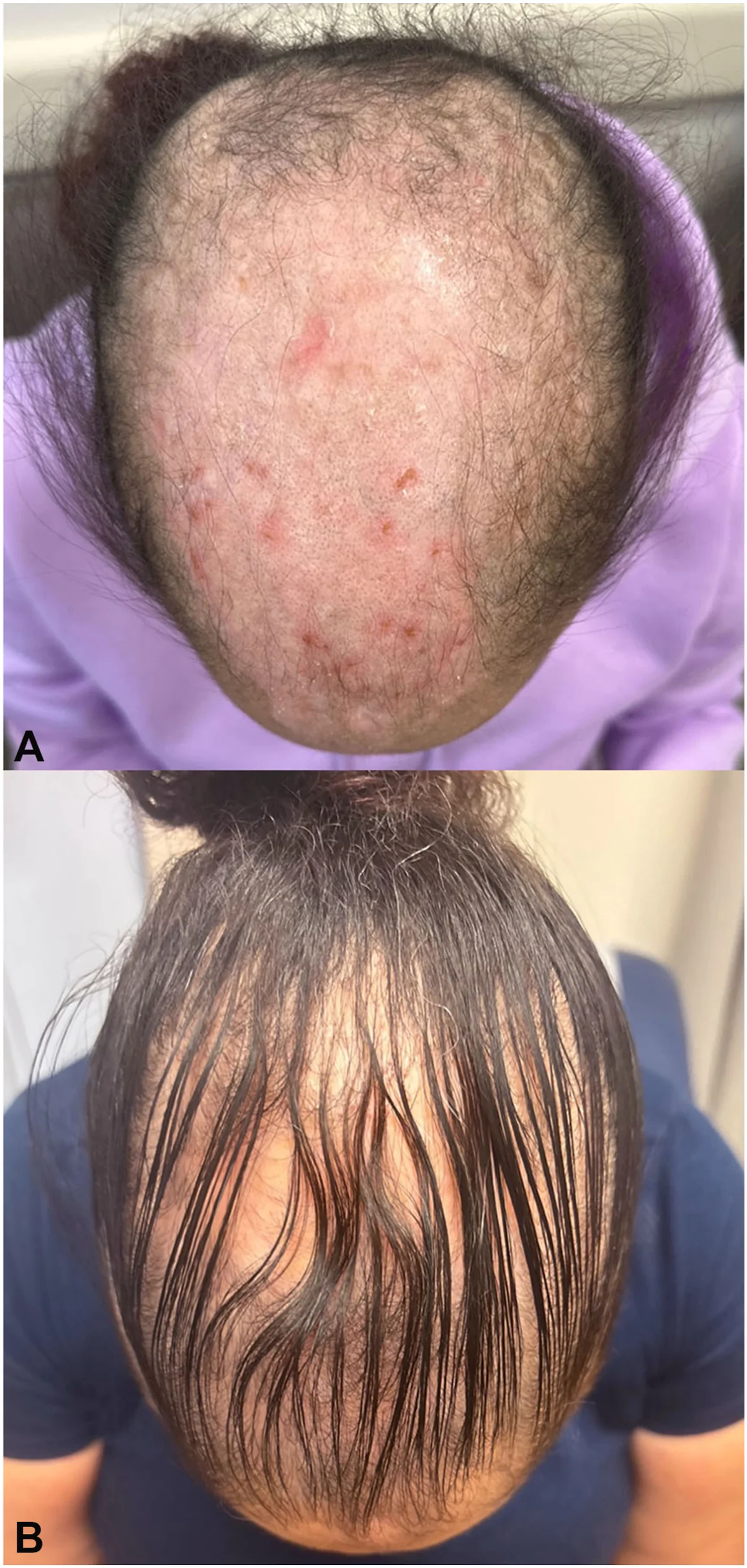
Scarring alopecia associated with cutaneous lupus before and after anifrolumab therapy. **A,** Patchy scarring alopecia prior to treatment. **B,** Notable hair regrowth following 7 months of anifrolumab infusions.

Given persistent disease despite multiple immunosuppressive therapies, intravenous anifrolumab 300 mg every 4 weeks was initiated in early December 2024, while hydroxychloroquine, mycophenolate mofetil, pentoxifylline, and sildenafil were continued. Marked improvement was observed after initiation of anifrolumab, with improvement in digital erosions, reduction in pain and swelling, and improved hand function. Prednisone was tapered from 40 mg to 5 mg, and no additional rituximab or belimumab infusions were administered.

By August 2025, her digital lesions had markedly improved (Figs 2, *B* and 3, *B*), and she no longer required topical corticosteroids or botulinum toxin injections. Scalp hair regrowth was also observed with adjunctive topical minoxidil therapy (Fig 4, *B*). She demonstrated improvement in her Cutaneous Lupus Erythematosus Disease Area and Severity index activity (CLASI-A) score from 32 before anifrolumab treatment to 8 after 8 months of therapy.^5^ At March 2026 follow-up, her CHLE remained improved and stable.

Notably, mild recurrence of symptoms occurred immediately prior to scheduled anifrolumab infusions and 2 missed anifrolumab infusions were associated with recurrent CHLE flares that improved after treatment was resumed. In April 2026, a greater than 6-week delay led to a severe CHLE flare, suggesting a treatment-dependent response. Overall, anifrolumab was well tolerated without significant adverse effects, and sustained clinical improvement across multiple sites has been maintained on ongoing therapy.

## Discussion

Dysregulated type I interferon (IFN) signaling is increasingly recognized as a central driver of vascular and immune-mediated injury in cutaneous lupus erythematosus. CHLE is believed to arise within a self-amplifying, IFN-rich microenvironment driven by dysregulated innate and adaptive immune responses.^6^ Molecular overlap between secondary chilblains and monogenic type I interferonopathies, including *TREX1*-associated disease, further supports a pathogenic role for type I IFN signaling in CHLE.^6^

Anifrolumab is a monoclonal antibody targeting the type I IFN receptor subunit 1 that has demonstrated significant improvement in cutaneous lupus activity in randomized trials of SLE.^7^ Recent case reports and a large multicenter real-world cohort further support the effectiveness of anifrolumab in refractory CHLE.^3^^,^^8^, ^9^, ^10^ In a national series of 115 patients, Muntaner-Virgili et al reported improvement in multisite refractory cutaneous lupus, including CHLE, with significant reductions in CLASI-A scores and corticosteroid use.^10^

Complementing these findings, our case further supports the potential efficacy of anifrolumab in a patient with severe, treatment-refractory CHLE. Initially presenting with ulcerative perniosis and scarring alopecia, the patient experienced sustained multisite clinical improvement, including resolution of digital erosions, hair regrowth in areas of prior scarring alopecia, a reduction in CLASI-A scores (32-8), and successful corticosteroid tapering. Importantly, recurrent disease flares following delayed anifrolumab infusions suggest a treatment-dependent response and that uninterrupted therapy may be particularly important in severe refractory CHLE.

We acknowledge that the patient's initial clinical improvement may have been influenced by IV methylprednisolone and rituximab administration during hospitalization. In addition, cytokine profiling was not performed, limiting our ability to correlate immunologic changes with the observed clinical response. However, the patient's CHLE had remained refractory for more than 5 years despite many of these therapies. Although rituximab and corticosteroids may have contributed to the initial improvement, the sustained clinical response over more than 16 months in the absence of additional rituximab administration supports the possibility of a treatment-dependent effect of anifrolumab.

This case supports type I IFN receptor blockade as a promising therapeutic strategy in refractory CHLE by demonstrating durable multisite improvement in severe, functionally impairing CHLE. Larger prospective studies are needed to define the durability of response, optimal maintenance intervals, and long-term safety of anifrolumab in CHLE.

## Conflicts of interest

None disclosed.
